# Improving eye care services for children

**Published:** 2024-02-09

**Authors:** Aeesha Malik, Clare Gilbert

**Affiliations:** 1 Clinical Assistant Professor and Consultant Paediatric Ophthalmic Surgeon: International Centre for Eye Health, London, UK.; 2 Professor of International Eye Health: International Centre for Eye Health, London School of Hygiene & Tropical Medicine, London, UK.

***I knew as soon as my son was born there was something wrong with his eyes***. Why child health must include eye health.

**Figure:**
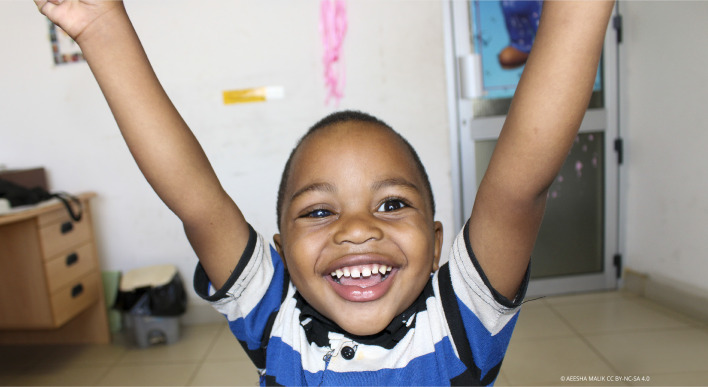
Good vision during infancy and early childhood is vital for normal development, including social development. tanzania

Ashura Hemed knew soon after her son, Shadrack, was born that there was something wrong with his eyes. They looked larger, were watery, and he hardly ever opened them. Ashura had visited the local primary level child health clinic five times over three months. Not knowing how to examine Shadrack, or what to look for, the child health care workers either ignored Ashura's questions and concerns, or gave her antibiotic eye drops that had no impact on Shadrack's condition.

Shadrack was lucky – his family happened to live near Dar es Salaam, the largest city in Tanzania, which has child eye care facilities. His determined mother took him to an eye department where he was diagnosed with congenital glaucoma. He is now doing well and is being followed up after surgery.

Shadrack was one of the lucky ones – if he hadn't been correctly diagnosed and treated in time, he would have grown up blind or severely visually impaired.

Parents all over the world face similar problems to Shadrack and his family. Primary eye care services for children are limited or non-existent in many low-income settings, particularly in rural areas, away from big cities. This means that children often present late to eye care services – sometimes many months, or even years after their parent or carer first noticed the problem. Delayed treatment can lead to worse vision and developmental outcomes than if treatment had been given on time. In the case of retinoblastoma (a form of eye cancer), delays can result in the death of the child.

Children are particularly vulnerable to the consequences of inadequate eye care. Good vision is needed from birth until the age of seven years to ensure that children's brains develop the visual pathways that will lead to healthy adult vision. Anything that obstructs their vision during this crucial time, such as cataract or corneal scarring, can result in lifelong visual impairment or blindness, as well as delays to their overall physical, mental, and social development. This is the case even if their eye condition is treated when they are older.

**Figure:**
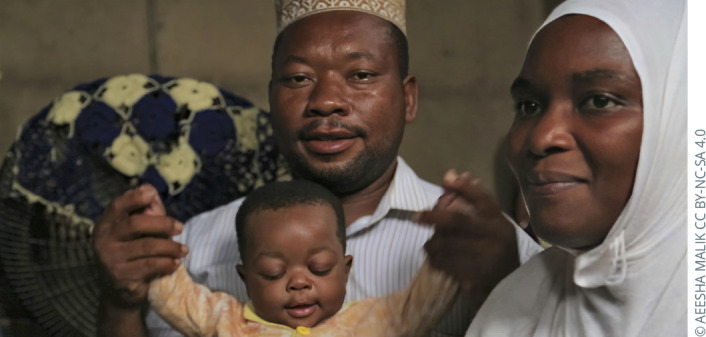
Parents with their baby, who has congenital glaucoma. tanzania

## Improving eye care services for children

To address the critical issue of children presenting late, there needs to be routine screening of children, at primary or community level, to detect eye conditions in time (page 8). However, there are only a few screening guidelines for eye conditions in children, and most are for high-income countries.^1^ One notable exception is India's guideline on universal eye screening in newborns, which includes retinopathy of prematurity.^2^

There has been some progress. Recently, the World Health Organization (WHO) included eye screening as part of the general examination of all newborns.^3^,^4^ However, screening guidelines are also needed for other conditions and in older children. Personnel who care for children at primary, secondary, and tertiary levels must receive the necessary training to detect, refer, and/or diagnose and manage children who need help.

**Figure:**
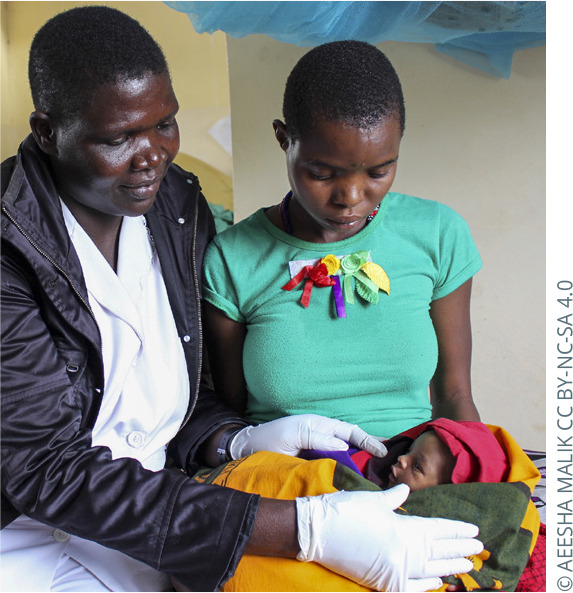
Good vision during infancy and early childhood is very important for normal development, including social development. tanzania

Children under the age of 5 years frequently – perhaps more so than at any other time in their lives – visit primary health care facilities, e.g. for vaccinations, growth monitoring, or when they are ill. Personnel working in primary health clinics are, therefore, very well placed to detect children with eye conditions and provide initial care, referring them if needed.

In 1995, WHO and the United Nations Children's Fund (UNICEF) jointly launched a global programme called the Integrated Management of Childhood Illness; when newborns were added, this became known as the Integrated Management of Newborn and Childhood Ilnesses (IMNCI) programme. IMNCI contains modules for the major conditions affecting children, such as fever or malnutrition, and each module has an algorithm that helps workers determine the severity of the condition and tells them how to manage it at primary level. More than one hundred countries have implemented the IMNCI programme to date. Although there is a module for ear problems, there is not yet one for eye problems.

## Tanzania: showing what is possible

During a pilot study in Tanzania, an IMNCI module for eye problems was developed in collaboration with the ministry of health.^4^ After successful completion of the pilot study, which demonstrated that eye health could be included in child health services in a way that was practical and acceptable to primary health care workers,^4^ the module was formally included in the national IMNCI policy. This resulted in more than 3,500 primary health care workers being trained in eye care. According to data from the ministry of health, these health workers see an average of 5.8 million children annually, potentially identifying nearly 250,000 children with eye problems every year.

The Tanzanian experience shows that it is possible for policy change to take place at national level, and for eye health to be included in a national child health strategy. This model can be applied in other countries, especially in the more than one hundred countries where IMNCI is already in place. More advocacy may be needed at WHO level for the inclusion of an eye care module in IMNCI; this may help to encourage more countries to follow Tanzania's example.

**Useful websites**

The Global Child Eye Health Consortium. https://tinyurl.com/y7bexr5

IMNCI project video, including the case study in this article: https://tinyurl.com/sbupb6kf

IMNCI training videos: https://tinyurl.com/bpamvcmt

Arclight training videos: http://tinyurl.com/CEHJ-arclight

IAPB School eye health guidelines: https://tinyurl.com/ypnja65y

Enabling good vision in early childhood is very important as it allows a child to develop normally and reach their full potential in every area of their life. Improving detection, referral, and treatment of eye conditions in children is therefore in line with a recent shift in global child health policies: from an emphasis on how to help children ‘survive,’ to a focus on how children can ‘thrive.’ This provides a major opportunity for the inclusion of eye health in IMNCI and other national and global child health policies for young children. Once policies are in place, appropriate training and equipment for the local context will also be needed.

Eye care providers on their own cannot solve the problem of child eye health. We need to reach out to those making child health policies and running child health programmes to ensure eye health is integrated into these policies and programmes. One possible step is to advocate for the inclusion of eye care into IMNCI programmes in your own country, using the Tanzania case study as evidence and a model. Another helpful step is to refresh your own knowledge of child eye care and share this with others. The articles in this issue were designed to be as accessible as possible, to as wide an audience as possible, so they can be easily used and/or adapted by those who teach others.

By working together with others who care for children, we can ensure that no child becomes, or remains, needlessly blind.
